# Supplementary figures and images for: Persistent thermal input controls steering behavior in Caenorhabditis elegans
Source: PLoS Comput Biol. 2021 Jan 8;17(1):e1007916. doi: 10.1371/journal.pcbi.1007916 (PMC7819614; doi:10.1371/journal.pcbi.1007916)

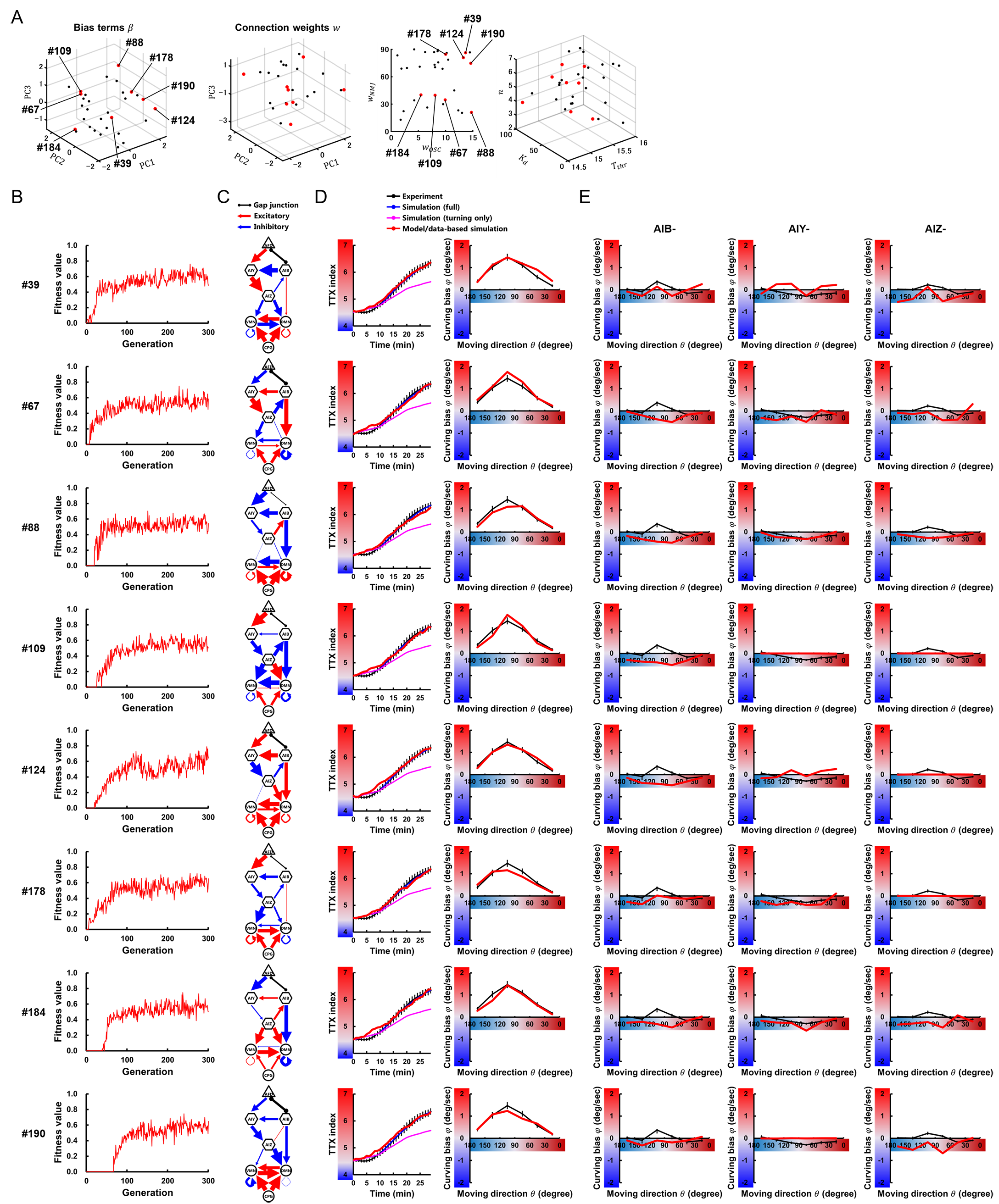

Supplement: S1 Fig — (A) 32 independent parameter sets having a fitness score of at least 0.5, including 8 parameter sets having a fitness score of at least 0.6 (B) are plotted in parameter spaces as black dots and red dots, respectively. The parameter subspaces are the space defined by principal components of 6 bias terms that shift sensitivity range of inter- and motor neurons (leftmost panel), the space defined by principal components of 12 connection weights of chemical/electrical synapses (second left panel), the plane defined by a connection weight from a pattern generator to motor neurons and a connection weight of neuromuscular junction (second right panel), and the space defined by 3 terms that determine response property of a thermosensory neuron (rightmost panel). Individual parameter sets were assigned numbers (#) from 1 to 200. For the 8 good models, the circuit diagram (C), the time course of TTX index, and the profile of curving bias (D) are plotted. In the circuit diagrams, thickness of each connection is represented proportionally to its connection weight. All the 8 models reproduced empirical impairments of curving bias upon ablating individual interneurons AIB, AIY, and AIZ (E). (TIF) [file pcbi.1007916.s001.tif]

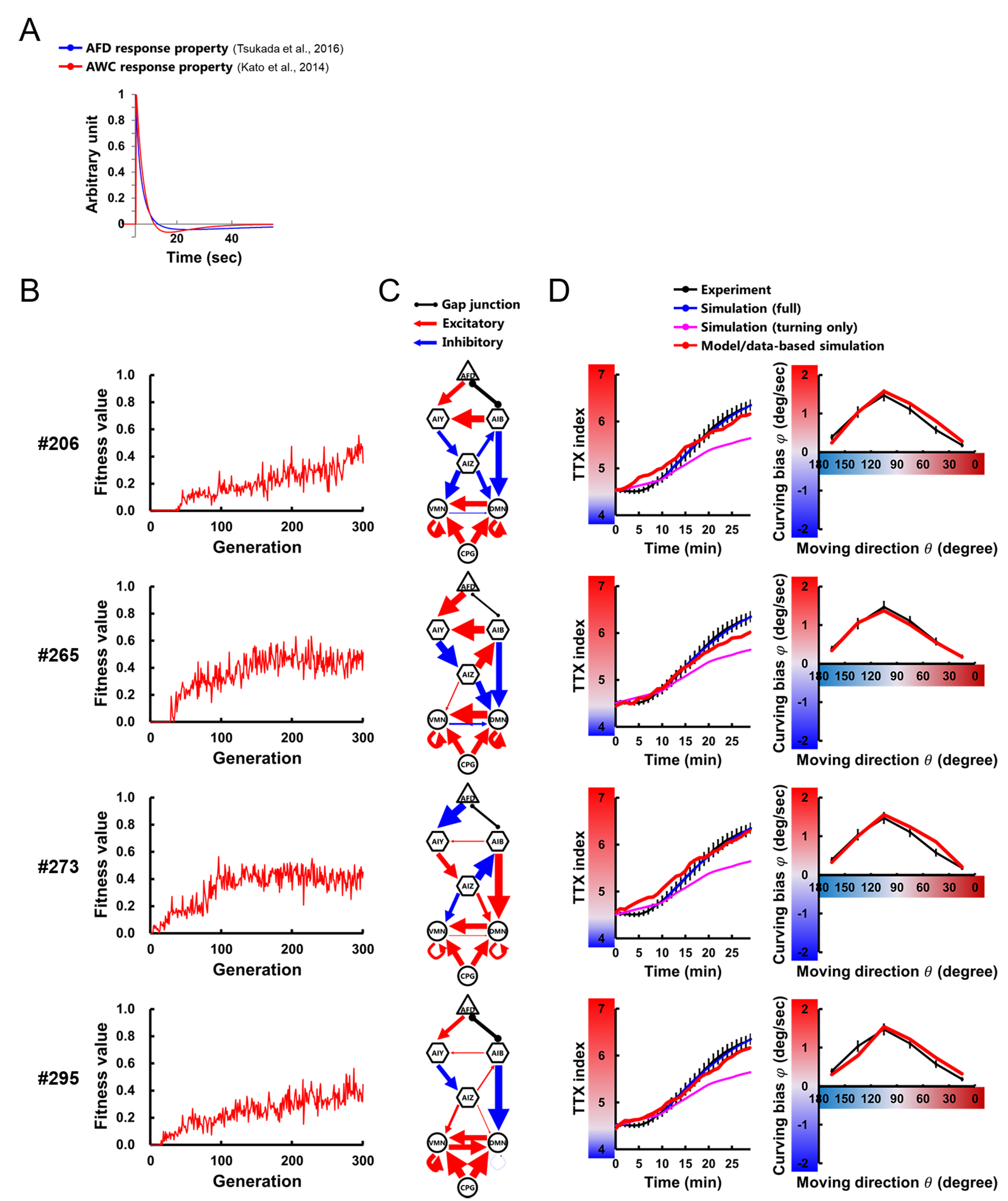

Supplement: S2 Fig — We performed 100 evolutionary searches in which the response property of AFD [18] was replaced with that of another sensory neuron AWC [23] (A), and 4 independent parameter sets having a fitness score of at least 0.5 were obtained (B). Individual parameter sets were assigned numbers (#) from 201 to 300. For the 4 good models, the circuit diagram (C), the time course of TTX index, and the profile of curving bias (D) are plotted. In the circuit diagrams, thickness of each connection is represented proportionally to its connection weight. (TIF) [file pcbi.1007916.s002.tif]

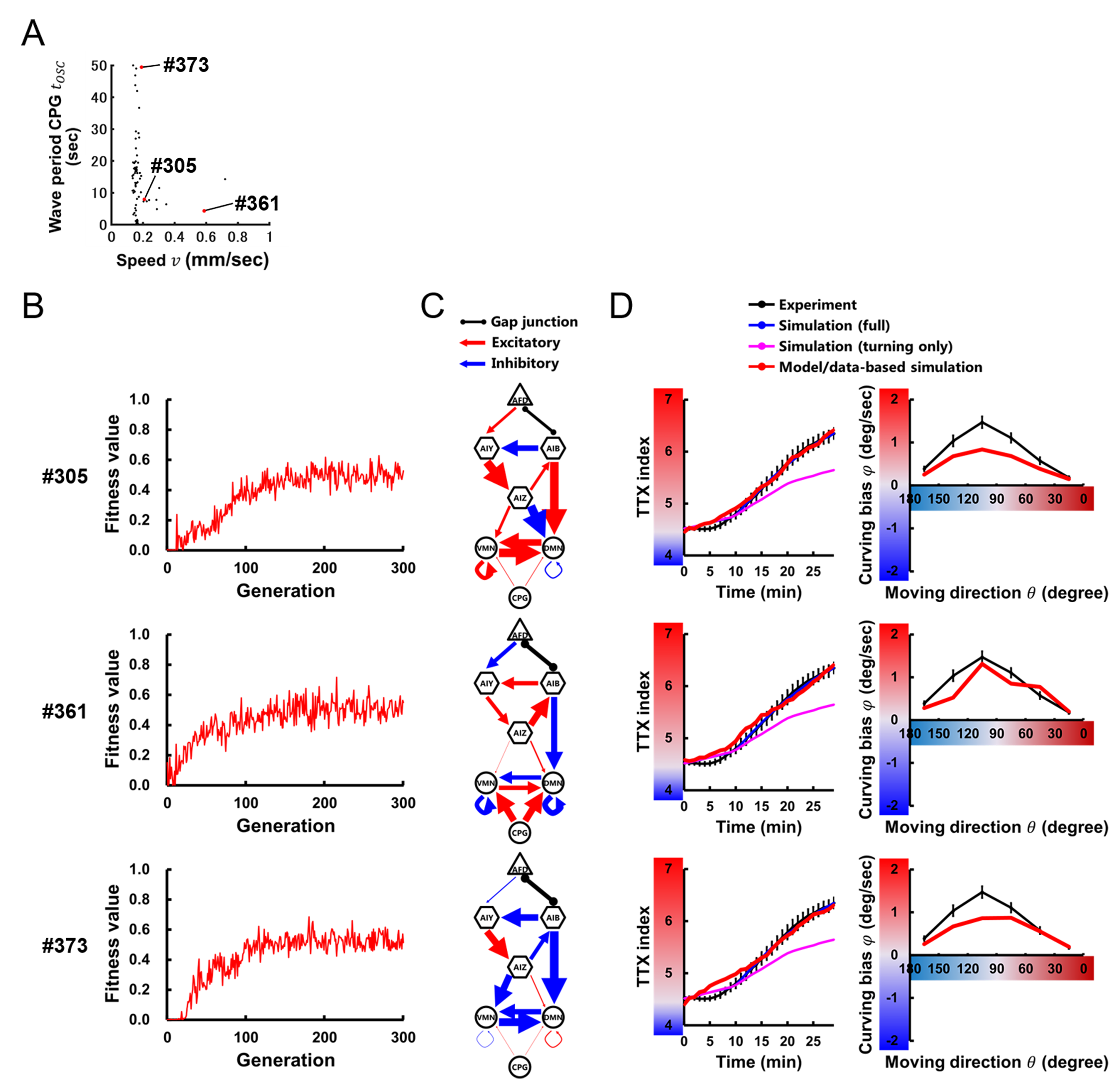

Supplement: S3 Fig — We performed 200 evolutionary searches in which moving velocity of model worms (v) and wave period of a pattern generator CPG (tOSC) were evolved, and 68 independent parameter sets having a fitness score of at least 0.5 were obtained (A and B). Individual parameter sets were assigned numbers (#) from 301 to 500. For the 3 representative models (red dots in (A)), the circuit diagram (C), the time course of TTX index, and the profile of curving bias (D) are plotted. In the circuit diagrams, thickness of each connection is represented proportionally to its connection weight. (TIF) [file pcbi.1007916.s003.tif]

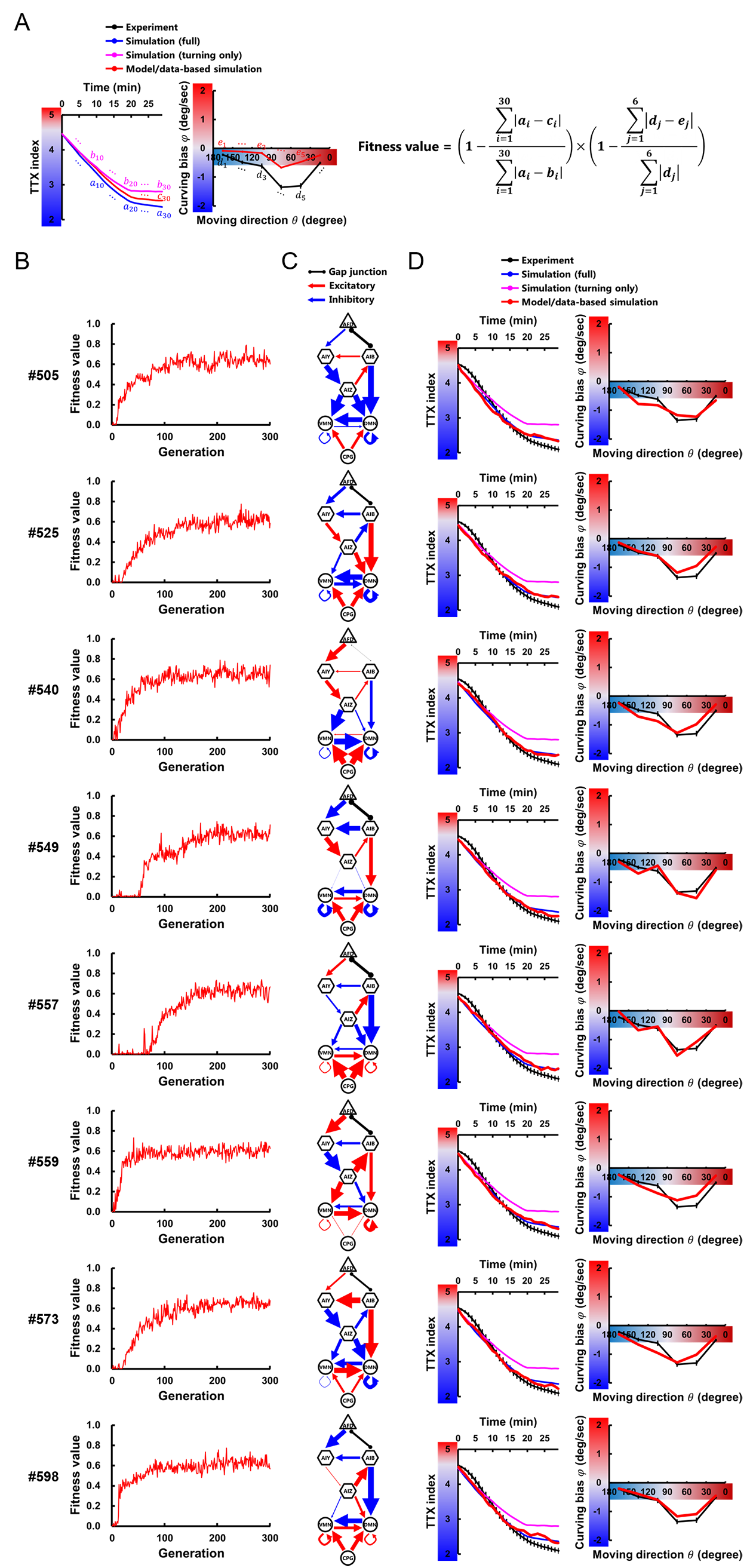

Supplement: S4 Fig — We performed 100 evolutionary searches in which the parameters were evolved to reproduce negative thermotactic behavior (A), and 8 independent parameter sets having a fitness scores of at least 0.6 were obtained (B). Individual parameter sets were assigned numbers (#) from 501 to 600. For the 8 good models, the circuit diagram (C), the time course of TTX index, and the profile of curving bias (D) are plotted. In the circuit diagrams, thickness of each connection is represented proportionally to its connection weight. (TIF) [file pcbi.1007916.s004.tif]

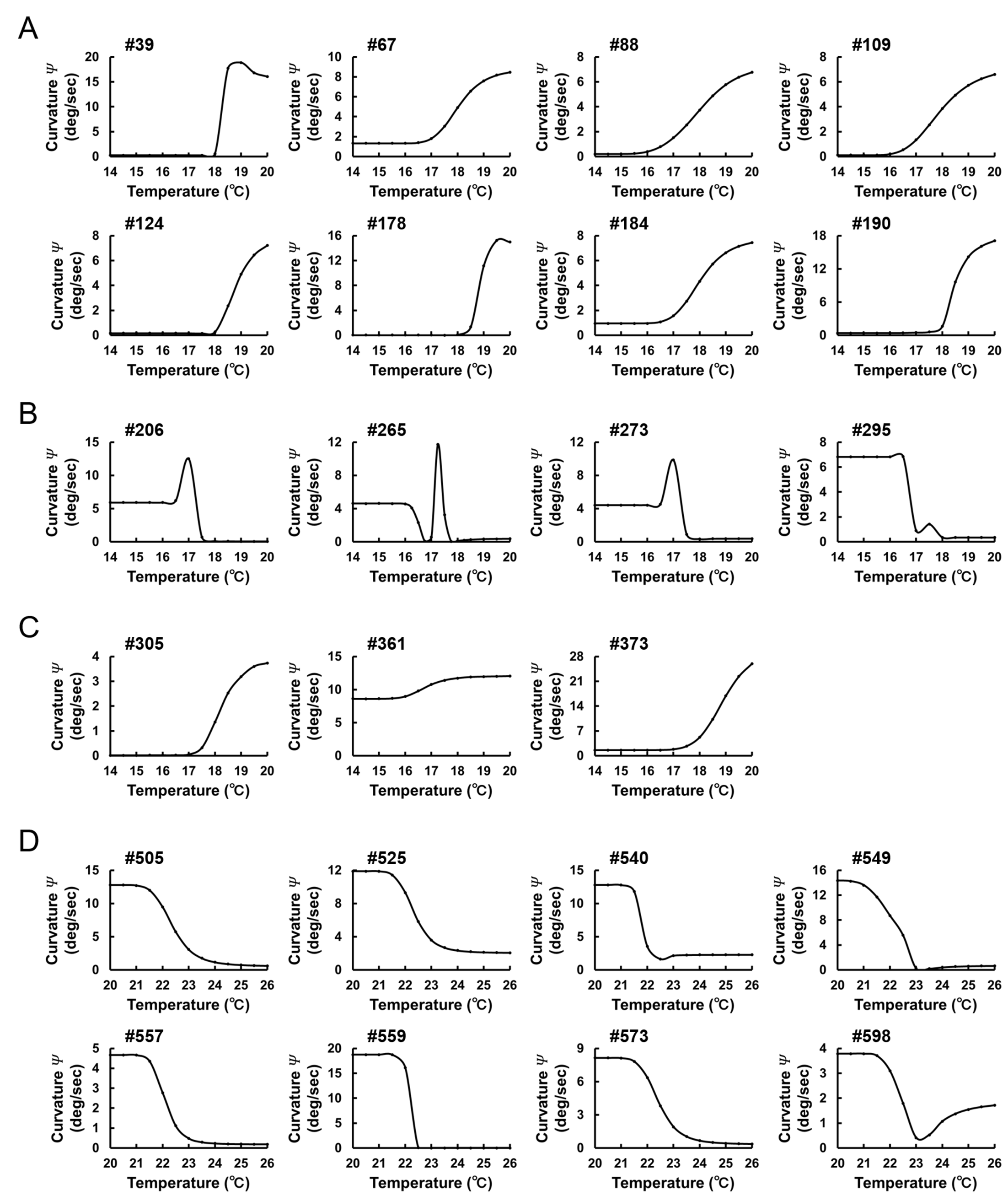

Supplement: S5 Fig — Steering curvature Ψ of the model worms were measured under the simplified simulation in which temperature of assay plates was set as constant (ranging from 14 to 20°C or from 20 to 26°C), and model worms were set not to perform turning. The parameter sets evolved through different evolutionary searches were employed for the simulation, and steering curvature Ψ of the individual model worms are plotted against temperature: (A) for S1 Fig, (B) for S2 Fig, (C) for S3 Fig, and (D) for S4 Fig. (TIF) [file pcbi.1007916.s005.tif]

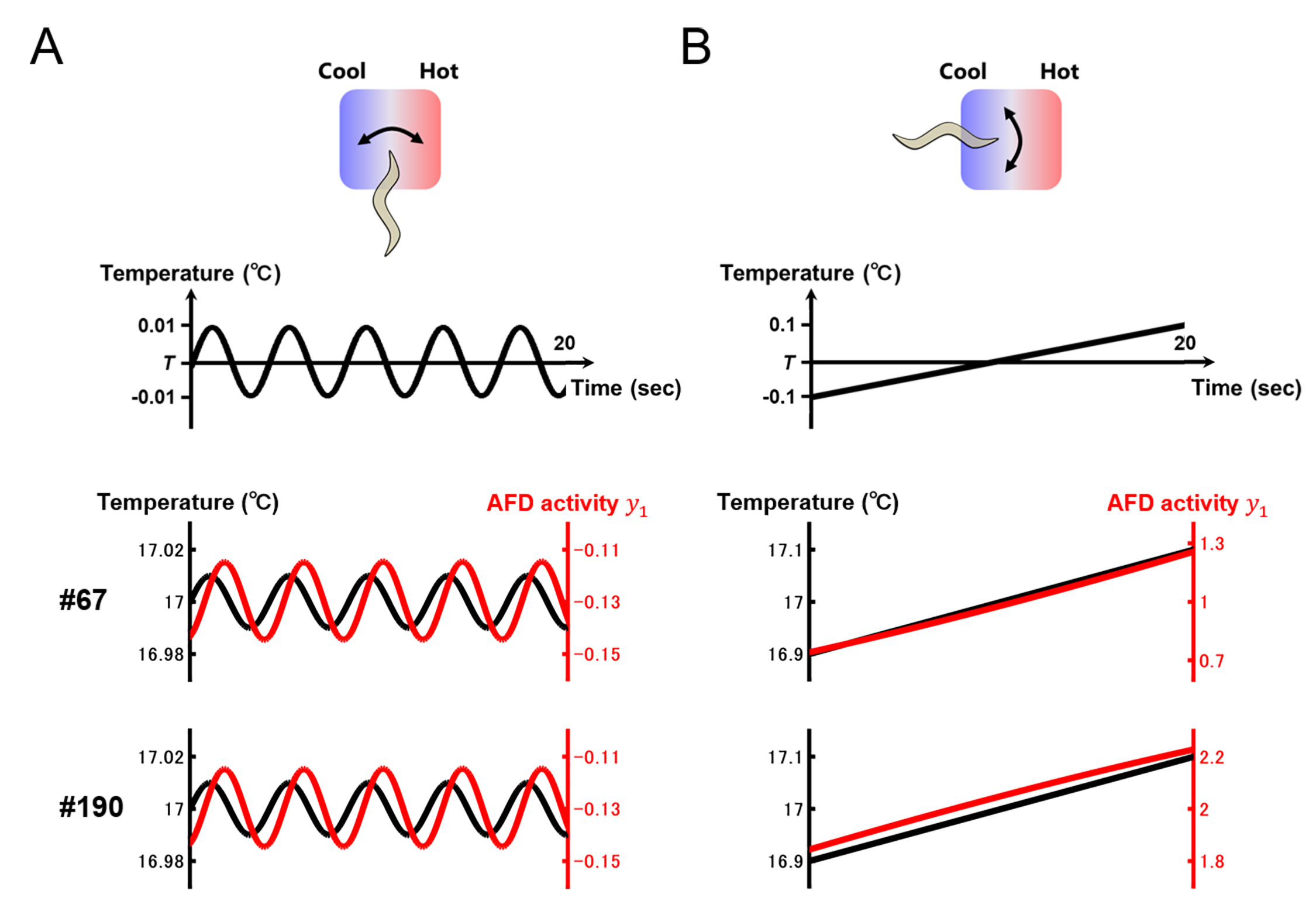

Supplement: S6 Fig — (A) Activity of AFD (red line) under temperature changes on the temporal scale of head swings (black line). Worms are assumed to be moving perpendicularly to a thermal gradient with their dorsal side heading toward warmer side. (B) Activity of AFD (red line) under temperature changes on the temporal scale of forward movement (black line). Worms are assumed to be moving straight up a thermal gradient. The simulation results with representative parameter sets (#67 and #190) are shown. (TIF) [file pcbi.1007916.s006.tif]

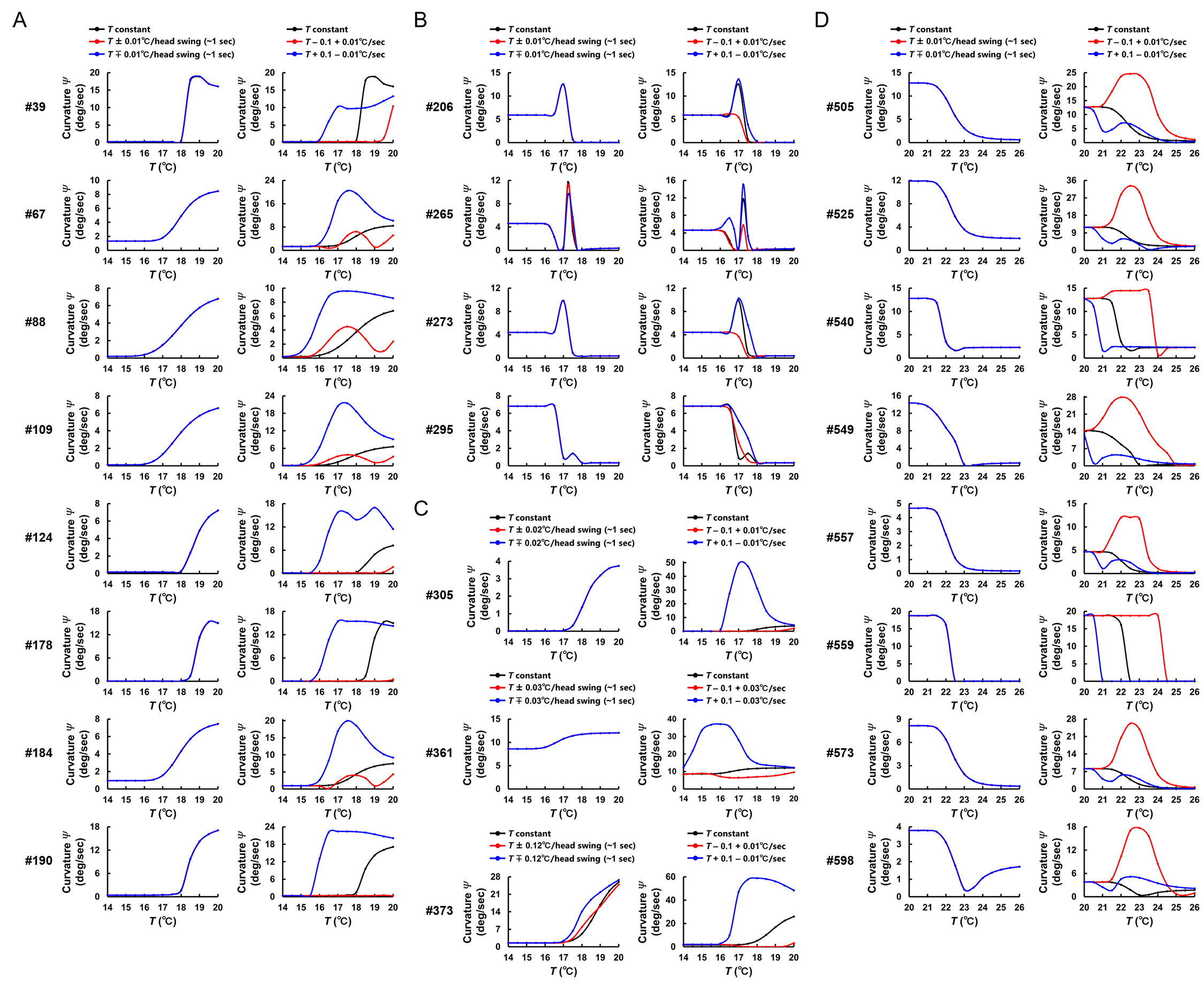

Supplement: S7 Fig — Steering curvature Ψ of the model worms were measured under temperature change on the temporal scale of head swings (left panels) and of forward movement (right panels). In the left panels, worms are assumed to be moving perpendicularly to a thermal gradient with their dorsal side heading toward warmer side (red lines) or colder side (blue lines). In the right panels, worms are assumed to be moving straight up a thermal gradient (red lines) or down a thermal gradient (blue lines). Ψ under these conditions were compared with those at the constant temperature (black lines). The parameter sets evolved through different evolutionary searches were employed for the simulation: (A) for S1 Fig, (B) for S2 Fig, (C) for S3 Fig, and (D) for S4 Fig. (TIF) [file pcbi.1007916.s007.tif]

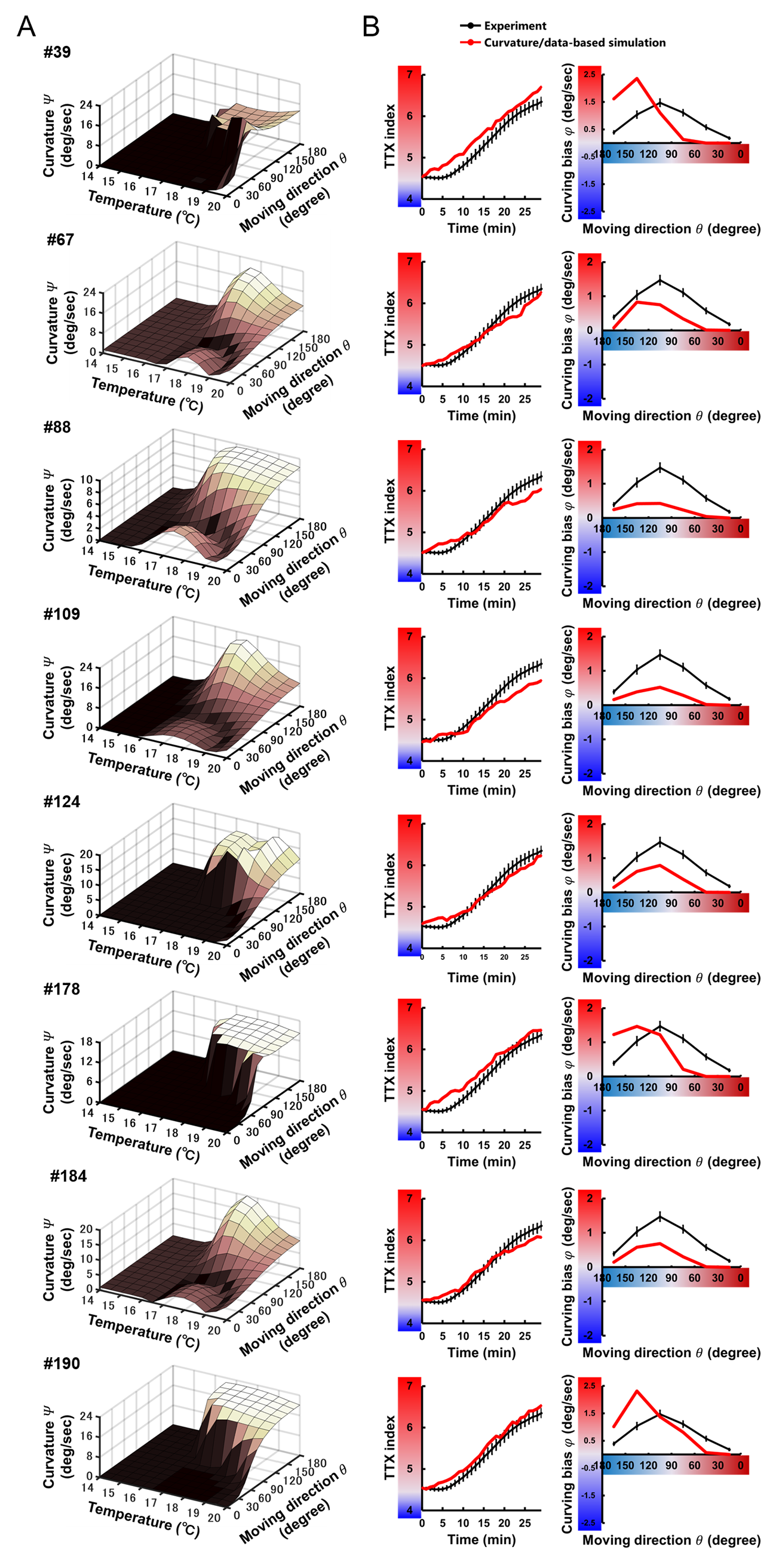

Supplement: S8 Fig — (A) Steering curvature Ψ were calculated and plotted against temperature and moving direction θ. (B) Plots of the time course of TTX index (left panels) and the profile of curving bias φ (right panels) in experiments (black lines) and simulations (red lines) in which the states of the worm are updated according to the empirical data for turning and to the profile in (A) for steering. (TIF) [file pcbi.1007916.s008.tif]

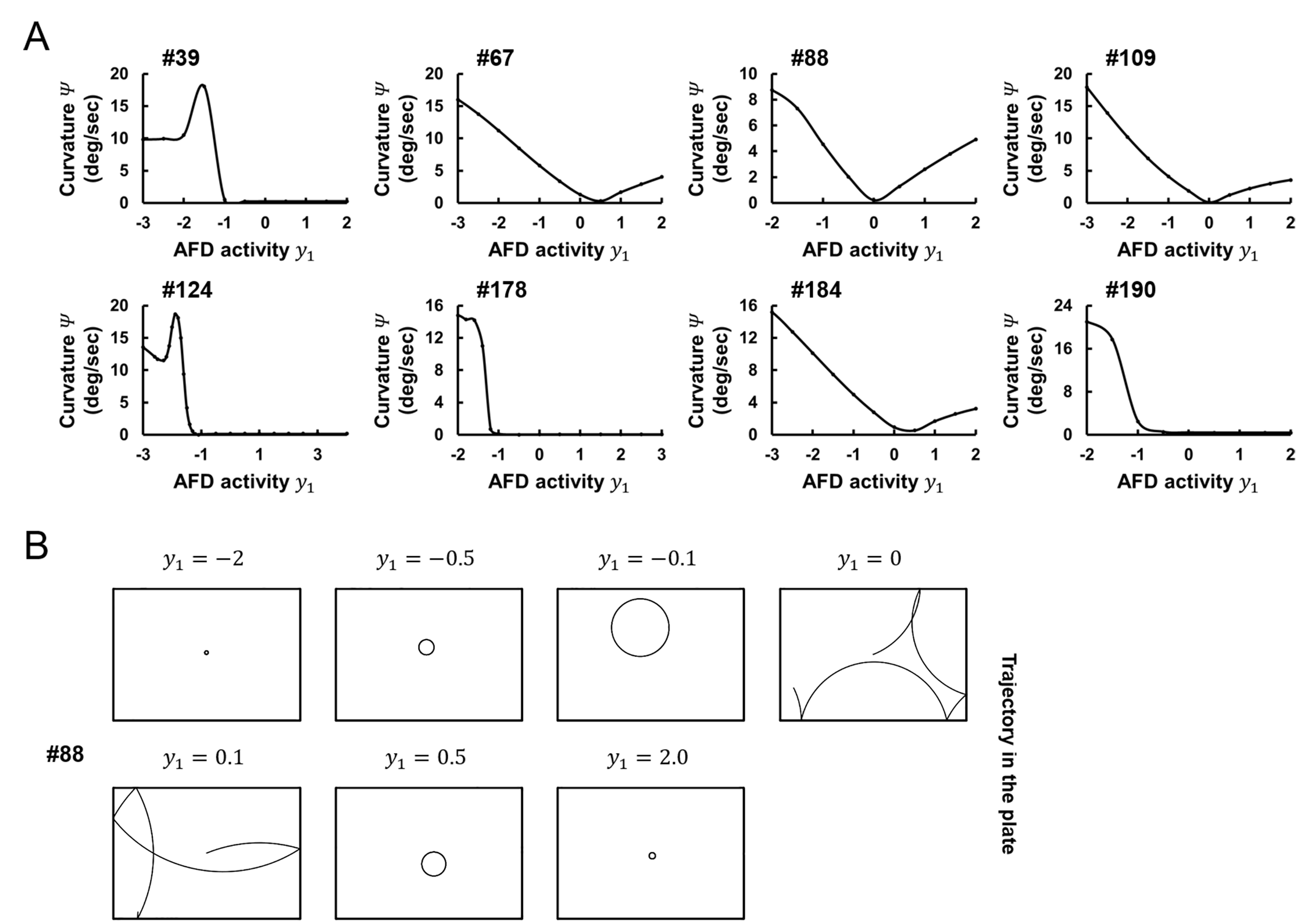

Supplement: S9 Fig — (A) Steering curvature Ψ of the model worms were measured under the simplified simulation in which AFD activity y1 of the model circuits was fixed at the constant values, and model worms were set not to perform turning. (B) Trajectories of the model worm with representative parameter set (#88) on the fixed AFD activities. (TIF) [file pcbi.1007916.s009.tif]

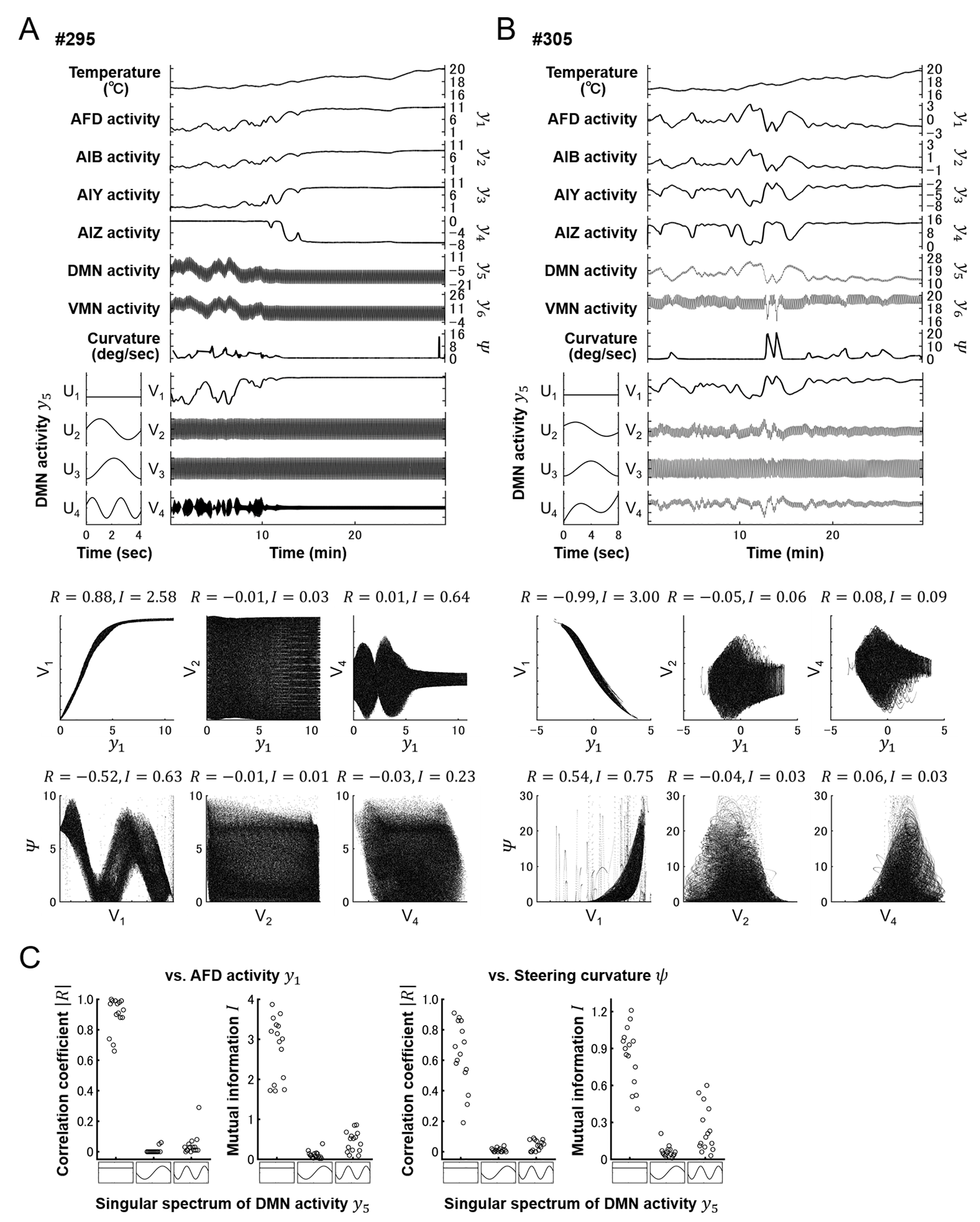

Supplement: S10 Fig — (A and B) Upper panels are time series of temperature sensed by the model worms evolved in S2(A) Fig and S3(B) Fig, activity of individual neurons y1−6, steering curvature Ψ of locomotion, first four singular spectrums U1−4 of dorsal motor neuron activity y5 decomposed by singular spectrum analysis, and their magnitude V1−4. Scatter plots of each variable in V1−4 versus AFD activity y1 or steering curvature Ψ are shown in lower panels, where correlation coefficients R and mutual information I among the time series are measured and plotted. (C) Plots of R and I measured among variable in V1−4 versus AFD activity y1 and steering curvature Ψ in all the 15 good/representative models evolved in S1–S3 Figs. (TIF) [file pcbi.1007916.s010.tif]

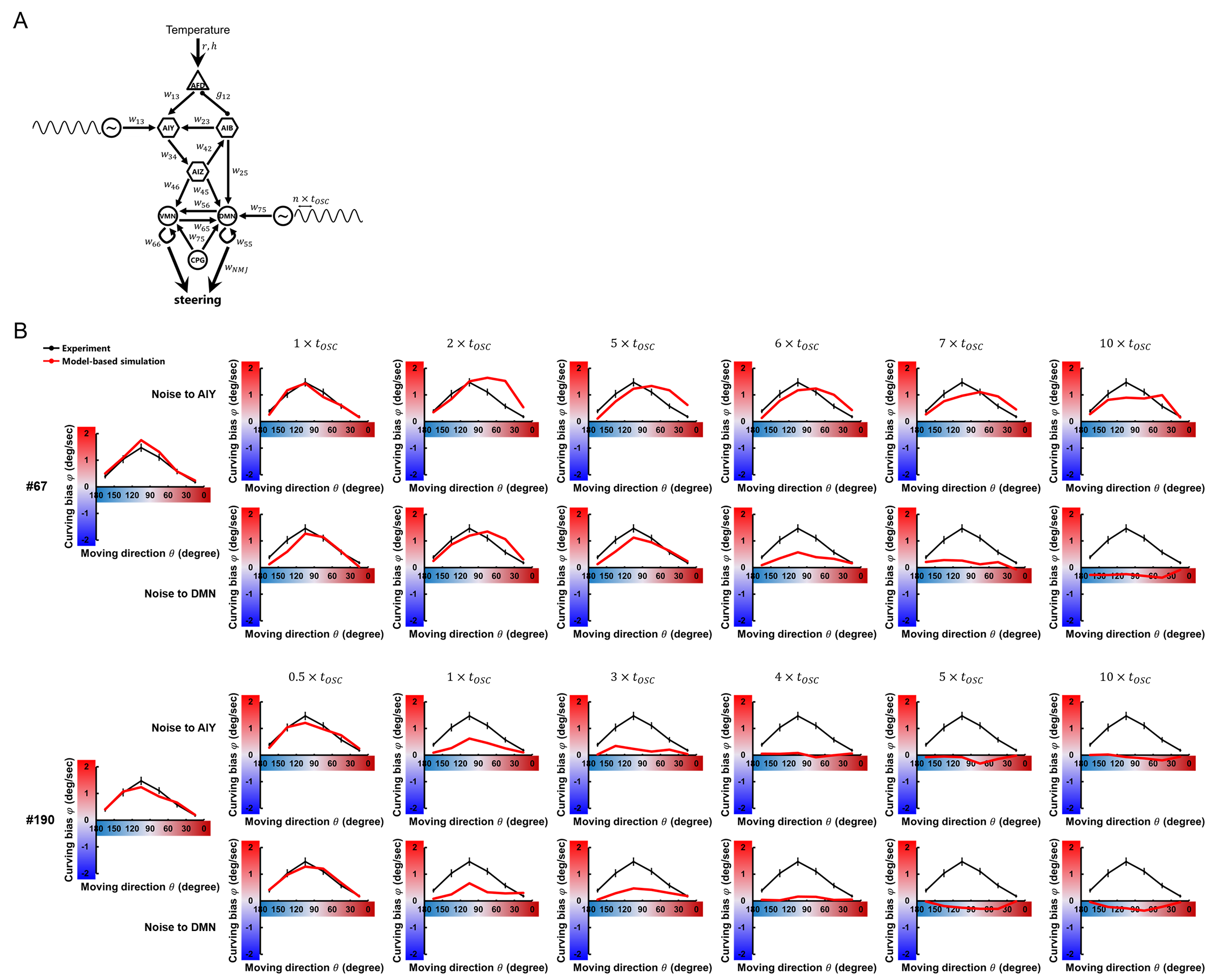

Supplement: S11 Fig — (A) A different time scales of noise component are added in the representative model circuits. The noise component is set to transmit oscillatory input to either AIY or DMN, and the wave period of the oscillation was changed from that of DV head bending (tOSC) to 10 times longer value. (B) Profiles of curving bias φ of the model worms under the applications of the oscillatory noise component to AIY (upper panels) or DMN (lower panels). (TIF) [file pcbi.1007916.s011.tif]

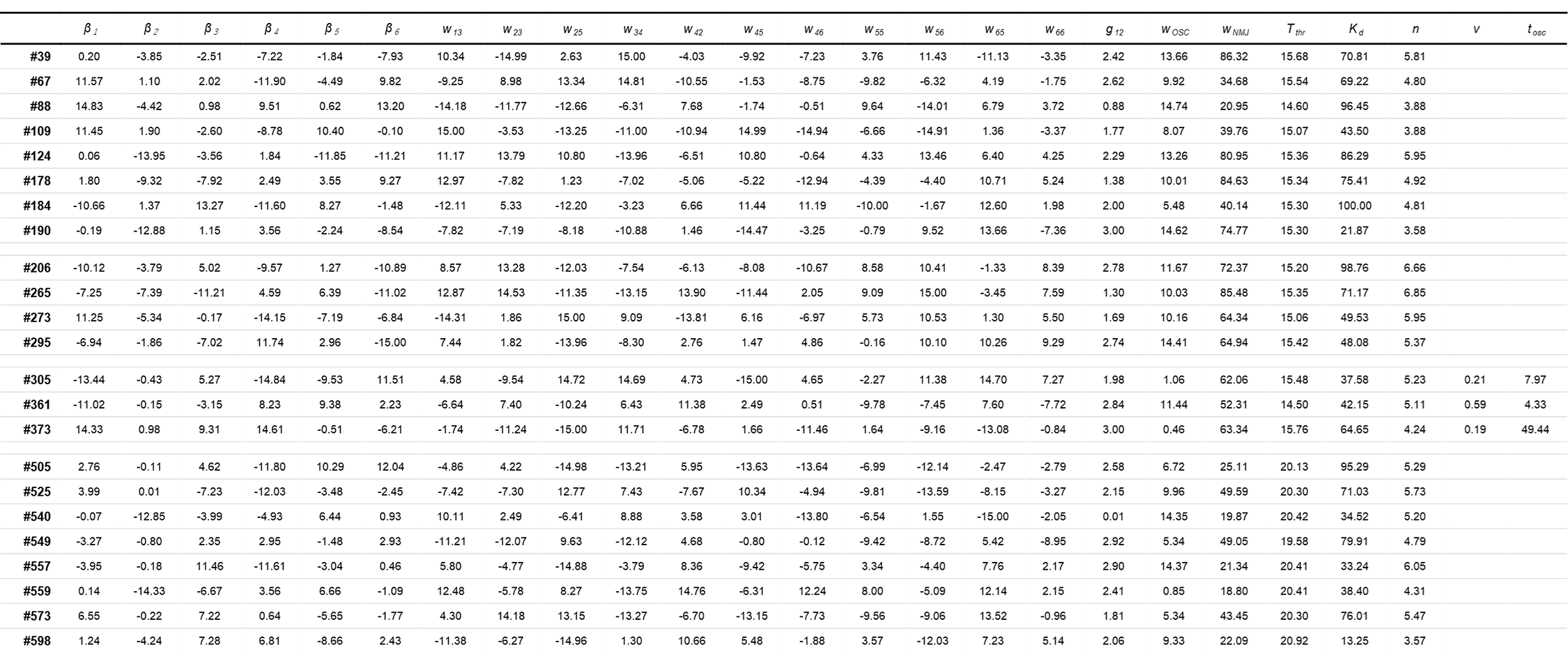

Supplement: S1 Table — Values of 23 (or 25) parameters in the 23 good/representative models evolved in S1–S4 Figs are listed. (TIF) [file pcbi.1007916.s012.tif]
