# Supplementary material for: Persistent thermal input controls steering behavior in Caenorhabditis elegans
Source: PLoS Comput Biol. 2021 Jan 8;17(1):e1007916. doi: 10.1371/journal.pcbi.1007916 (PMC7819614; doi:10.1371/journal.pcbi.1007916)
Supplement: Attachment — Submitted filename: Ikeda etal review 2020.pdf [file pcbi.1007916.s013.pdf]

In this study, Ikeda and colleagues used an evolutionary algorithm (EA) to identify neural network models that can drive sensory-gradient-guided steering behavior in simulated agents. The biological phenomenon that motivated this study was the thermotaxis behavior of *C. elegans*, which employs two key strategies: biased random walk (klinokinesis, akin to tumble-and-run in bacteria chemotaxis) and gradual steering during forward locomotion (klinotaxis). The authors used results from several experimental studies (in particular Ikeda et al. and Tsukada et al.) and the *C. elegans* connectome (White et al. 1986) to impose *a priori* constraints on the size, topology and functional properties of the model networks. Through *in silico* evolution, the authors identified 9 networks that yielded satisfactory fit to the thermotactic steering behavior in real animals. By examining these models, the authors concluded that the sole sensory neuron in the network, modeled after the thermosensory neuron AFD, functions by modulating the absolute magnitude of the curving rate, and does so over a timescale longer than that of the oscillatory locomotion of the (model) animal..

Overall, I found the approach of this study, namely analyzing computationally evolved networks in order to derive biological insights, quite interesting. However, I feel that the study could improve significantly on two fronts: 1) Provide a more mathematically precise and biologically motivated formulation of the network model and agent-based simulations. Importantly, if the goal of the study is to understand the circuit basis of steering/klinotaxis behavior, then the authors need to convince the reader that their evolutionary algorithm specifically targets the steering behavior, as opposed to generating alternative implementations of the biased random walk/klinokinesis. 2) The authors need to provide more substantial support for their main finding, namely that thermosensory inputs were transmitted from AFD to the downstream neurons at timescales slower than head oscillations (<=4 s per cycle). In particular, the authors need to convince the reader that this observation does not simply reflect the design of the thermal response kernel in AFD, which performs a temporal difference operation over a broad (~10 s) window.

SPECIFIC COMMENTS ON MODEL DESIGN/SELECTION:

1. I am not fully convinced that the evolutionary algorithm specifically targets the steering/klinotaxis behavior. According to the authors, models were selected based on goodness-of-fit to two key metrics: a) the temporal progression of the thermotaxis (TTX) index, b) the dependency of the absolute magnitude of the curving rate (φ) on movement direction. Firstly, since real animals perform both klinokinesis and klinotaxis during thermotaxis (Fig. 3C-E, Ikeda et al. 2020), the TTX index depends on a combination of these two behaviors. Since the simulated animals were also allowed to perform klinokinesis, it is unclear how much of the TTX profile result from klinokinesis alone. To specifically select for efficient klinotaxis behavior, the authors may need to reconsider the use of the TTX profile as a criteria. Secondly, a hall mark of klinotaxis behavior is the dependency of the *exact* curving rate on movement direction, which appears as a sinusoid-like function over a 2π period (see Iino and Yoshida, J Neurosci 2019). The authors have somehow chose to investigate the absolute value of the curving rate ($|\varphi|$). One risk looking at $|\varphi|$ instead of φ is that its dependency on heading direction, which shows a peak at $\pi/2$, closely resembles the relationship between reversal (where $|\varphi| \geq 150°$) and heading directions (Fig. 3C, Ikeda et al. 2020). Thus, without putting a cap on $|\varphi|$, the evolutionary algorithm could potentially select models that excel at klinokinesis instead of klinotaxis. I could not find in the manuscript a pre-

determined upper threshold for $|\varphi|$. I wonder what measure the authors have taken to ensure the selection is directed specifically at the klinotaxis behavior.

2. In addition to the above, there is further confusion on what $|\varphi|$ really is. Extrapolating from Fig 1D, $|\varphi|$ is the absolute value of the change in the movement direction θ, taken every 1s. Is $|\varphi|$ calculated the same way in the model? While the periodicity of the head oscillation was not explicitly defined in the manuscript, it seems to be around 2-4 seconds based on Fig 8. This would explain why $|\varphi|$ is oscillatory, as shown in Fig 7 and 8. However, in Fig 4-6 and many of the supp figures, $|\varphi|$ does not oscillatory when plotted against AFD activity, temperature and/or heading direction. Is "$|\varphi|$" in these latter cases a smoothed version of the instantaneous $|\varphi|$? I see two associated terms "curving rate" and "curving bias" in the paper. Could the authors define them explicitly in the Methods?
3. In describing their network model and agent simulations, the authors cite a number of previous studies without laying out the full set of equations and parametrizations (evolved or pre-defined). It would be greatly helpful to the reader if the author could present the full mathematical formulation of their model and agent simulations in the Method section. Examples of some questions I had while reading the methods:
    a. Which free parameters go into the nonlinearity h for AFD? Assuming it is a sigmoid function, is the center of the sigmoid (shown at 17°C in the schematic) an evolvable parameter? Is so, is it surprising the all evolved models have a sharp drop in curving rate at around 17°C (Fig. S2)?
    b. How are experimental data incorporated into the thermotaxis simulations? I assume that transitions rates between runs and various types of sharp reorientations were estimated from experimental data. Then what about in model ablation experiments shown in Fig. S1? Were the state transition rates taken from experiments on wild type animals or neuron-ablated animals?
    c. In the caption of Fig 2 (starting line 547), the authors state that "TTX index and curving bias from the simulations (red lines) and the experiment (black lines) were subtracted by those from the experiment without thermal gradients (gray lines)." Is it true that the experimental no-gradient TTX profile were subtracted from the simulated on-gradient TTX profile? Is it more appropriate to subtract a simulated no-gradient TTX profile instead?

COMMENTS ON THE ANALYSES AND INTERPRETATION OF THE EVOLVED MODELS:

1. The main finding of the paper was derived from the analyses in Fig 8, which shows that AFD activity co-varies with a component of the curving rate that varies on the timescales of >4 seconds. As mentioned earlier, it is unclear if this phenomenon simply results from the difference in the time scales of the AFD thermal response kernel r and the frequency of the head oscillation. Could the authors perform *in silico* experiments to test this possibility?
2. As mentioned above, the defining feature of klinotaxis is the animal's ability to gradually modify their heading direction to point up (in the context of positive chemotaxis) the gradient direction. By definition, the sign of the curving angle is crucially important, because it could either orient the animal towards or away from the desired movement direction. In contrast, the selected models were evolved to perform a gross modulation

of the magnitude of the curving rate $|\varphi|$ based on heading direction and they indeed do so. As the authors pointed out in Discussion, it seems that the solution these models arrived at is one of klinokinesis instead of klinotaxis, whereby the agents vigorously reorient themselves until settling on a trajectory that efficiently brings them up the thermal gradient. I think this relates to my earlier comment that the evolutionary algorithm may not be specifically targeting the steering behavior (for which the sign of φ is crucially important), but may have developed a slower version of the biased random walk/klinokinesis. This distinction is important if the authors wish to propose that they have identified a potentially novel strategy for thermotaxis.

3. In line 197, the authors claim as a new finding that "Activity of a thermosensory neuron decides curving rates of locomotion**".** Since AFD is the sole sensory neuron in the model network, and given that the models were selected based on their ability to modulate curving rates, wouldn't it be obvious that any temperature-dependent modulation of the curving rate has to come through AFD?
4. Lastly, the dynamics of neural activity in Fig 7 and of the curving rate decomposition in Fig 8 were plotted as heat maps, which were hard to decipher. I would suggest replotting them as simple line plots.
