# Supplementary material for: Persistent thermal input controls steering behavior in Caenorhabditis elegans
Source: PLoS Comput Biol. 2021 Jan 8;17(1):e1007916. doi: 10.1371/journal.pcbi.1007916 (PMC7819614; doi:10.1371/journal.pcbi.1007916)
Supplement: Attachment — Submitted filename: ResponseToReviewers_20201011.docx [file pcbi.1007916.s014.docx]

*PLOS Computational Biology*

Editorial Board

Oct 11, 2020

Dear Dr. Ahamed and Dr. Graham,

Thank you very much for your letter regarding our manuscript entitled “**Persistent thermal input controls steering behavior in** ***Caenorhabditis elegans***” (**PCOMPBIOL-D-20-00676**). We are pleased to see that the reviewers were positive and constructive about our manuscript.

As described below, we have conducted additional analyses and rewritten the manuscript to address the concerns raised by the reviewers. The comments by the reviewers are attached in *italic* style, and our point-by-point responses follow. In the manuscript, revised parts are indicated as red letters. We believe that our responses and revisions address all the concerns in our previous manuscript, thereby making the manuscript suitable for publication by *PLOS Computational Biology*.

Sincerely,

Muneki Ikeda, Ph.D.

Postdoctoral Scholar, Department of Neurology,

University of California San Francisco

E-mail: muneki.ikeda@ucsf.edu

**Responses to the comments from Reviewer #1:**

*The authors of this manuscript used neuroanatomical information available for the temperature sensing and response circuit in the nematode C. elegans to produce a (empirically-informed) model for thermotaxis in C. elegans. They use evolutionary searches to find patterns of connection strength and sign between the AFD, AIB, and AIY neurons (among others) capable of modeling observed steering rates. The authors found that steering rates were modulated by inputs at scales consistent with forward locomotion (rather than the faster oscillatory head swings).*

*I thought that the manuscript was well written and that the results presented supported the conclusions drawn. The information contained in this effort should prove useful to scientists interested in sensory integration in general, and thermotaxis in particular. I have a few comments that are mostly directed to the framing of the work, rather than its design and performance.*

*1) The work undertaken is restricted to animals migrating up a thermal gradient towards a cultivation temperature (except for the isothermal experiments). However, work on C. elegans on animals escaping noxious temperature stimuli, or down thermal gradients towards cultivation temperatures. Did the authors attempt these* *scenarios and how do they results reconcile with those behavioral scenarios?*

To investigate the different scenario from migrating up a thermal gradient, we performed additional evolutionary searches in which model animals were evolved to migrate down a thermal gradient and steer toward lower temperature (new **S4A Fig**). In our previous work, steering behavior for migrating down a gradient was shown to be mediated by similar set of neurons with that in **Fig 2A** (Ikeda et al., 2020). We therefore employed the same model circuit and evolved the same set of parameters as in the scenario of migrating up. Across 100 searches, we obtained 8 independent parameter sets having a fitness score of at least 0.6 (new **S4B Fig**) and confirmed that the equivalent mechanism was implemented for steering behavior; the steering curvature of model worm’s trajectory was larger (or smaller) as the temperature rises (or falls) persistently (new **S7D Fig**). This result supports that our result is not restricted to the scenario of migrating up. We added above results in the section 2, paragraph 2 of Results (new page 8, line 182–186).

In the scenario of escaping behavior from noxious temperature, another thremosensory neuron AWC is shown to contribute (Kotera et al., 2016; Ikeda et al., 2020), and therefore different mechanism for steering behavior is potentially underlying. We would like to leave this scenario for our future study.

*2) The idea that* *longer time integration is preferred seems plausible for an organism that locomotes through sinusoidal waves such as C. elegans. Is there any thoughts on the* *interaction between sideways and forwards locomotion in the production of the model? For example, if younger (smaller) worms (with different sinusoidal excursion and forward velocity) were used, would the plots on Fig 4 B and C now look different? In other words, would the model scale across animal sizes, or would you predict it to be best suited across specific forward velocity and head bend frequency ranges?*

To investigate the interaction between sideways and forwards locomotion in the model worms, we performed additional evolutionary searches in which moving velocity of model worms ($v$) and wave period of a pattern generator CPG ($t_{OSC}$) were evolved within the range $\left[ 0.01, 1 \right]$ mm/sec and $\left[ 0.5, 50 \right]$ sec, respectively. Across 200 searches, we obtained 68 independent parameter sets having a fitness score of at least 0.5. As plotted in new **S3A Fig**, a large fraction of the evolved $v$ was approximately 0.2, close to the empirical value, whereas the evolved $t_{OSC}$ did not show preferred values. This result implies that the specific magnitude of thermal input sensed through forward locomotion is preferable for reproducing thermotactic behavior. By contrast, thermal input sensed through head bend is not mainly employed even under these slower sinusoidal excursions. These implications were supported by the plots in new **S7C Fig**, though steering curvature of the model with slower head bends (#373) was slightly modulated upon oscillatory thermal input. Taken together, we predict that the mechanism for thermotactic steering behavior would be conserved across scales of worm size: younger worms and longer worms (*lon* mutants). We also predict, however, even larger animals such as *Drosophila* larva might be executing thermotactic steering based on larger thermal input sensed through its head casting (Luo et al., 2010). We added above results and discussions in the section 2, paragraph 2 of Results (new page 8, line 179–182) and in the paragraph 2 of Discussion (new page 12–13, line 293–295).

*3) The authors suggest that in addition to using klinotaxis for chemical orientation, worms use klinokinetics for thermal orientation. Could they please discuss the advantages and limitations for each sensory modality to the task of navigation, in a way that explained how each would necessitate its own behavioral mechanism?*

The difference between chemical orientation and thermal orientation would be reasonable when considering distribution profiles of each sensory modality in natural environment of *C. elegans*. Chemical attractants such as diacetyl and isoamyl alcohol are metabolic byproducts secreted by soil bacteria (worm’s food), and thus chemical gradients in natural environment are assumed to be bell-shaped distributions whose peaks contain rotting organic matter. Such distributions would be steep and thus provide large difference in chemical input sensed through head swings, which would be sufficient to evoke large neural responses to execute steering behavior. By contrast, thermal gradients in soil environment are assumed to be relatively uniform; temperature depends on the depth from soil surface. Therefore, the signal-to-noise ratio would be smaller, and the longer time integration of thermal input would be necessary for executing steering behavior. Above discussion is supported in our recent study (Dahlberg and Izquierdo, 2020) and added in the paragraph 2 of Discussion (new page 12, line 288–293).

On the other hand, however, we are assuming the possibility that the chemotactic steering behavior is also implemented klinokinetically. Although previous studies of neuroimaging (Hendricks et al., 2012; Liu et al., 2018) and optogenetics (Kocabas et al., 2012) suggest that worms could adjust the amplitude of head swings and thus klinotactically steer, the possibility of klinokinetic steering has not been tested. The implementation of klinokinetic steering during chemical orientation would be potentially verified in assays in which chemical stimuli are applied temporally, and worm behavior is monitored simultaneously, as performed in **Fig 4**. We added above discussions in the paragraph 3 of Discussion (new page 13, line 296–305).

*Smaller points:*

*1) The meaning of the boxes on the right side of Figure 6 or the right panel A of Figure 4 are only clear after reading the caption to Figure S5B. I would make the* *caption on these figures clearer.*

We added the caption “Trajectory in the plate” in new **Fig 4A**, **Fig 6A**, and **S9B Fig**.

*2) In Figure 7B why is the correlation between AFD and DMN and VMN not symmetrical? Is there a dorsal or ventral bias?*

Asymmetricity in DMN and VMN of the model comes from two factors. Firstly, they are innervated by a distinct set of interneurons; AIZ innervates both DMN and VMN, while AIB innervates only DMN (**Fig 2A**). Secondly, asymmetricity in connection weights (**S1C Fig** and **S1 Table**) leads to different responses in DMN and VMN. Especially, difference in weights of reciprocal connections (DMN⇄VMN) and recursive connections (DMN→DMN/VMN→VMN) would be largely contributing to the dorsal and ventral asymmetricity.

*3) I found Figure 8 is confusing/challenging (perhaps due to my unfamiliarity with the algebra). I think that from the perspective of a wider audience this figure could be further digested for the reader (perhaps in a supplementary figure).*

To make **Fig 8** easier to understand, we moved old **Fig 8D** to new **S10C Fig**. Also, we replaced the heatmap plots in old **Fig 8B** with the line plots. To support the understanding of **Fig 8A**, we described the mathematical formulations of singular spectrum analysis (**Eqs 11 and 12**) in the section 5 of Materials and Methods (new page 19, line 453–463) We hope these modifications have made **Fig 8** easier to digest.

*4) Line 43: C. elegans does not possess a 302-neuron brain. Rather, its entire nervous systems is comprised of 302 neurons.*

We replaced the word “brain” with “nervous system” (new page 3, line 45).

*5) The algorithms used and the raw data generated (both from the behavioral experiments as well as the modeling data) was not deposited in an accessible repository (although the supplement does provide graphical summaries). I am not sure if the journal does or not require these to be submitted.*

We now deposited all the codes and used for this study in the GitHub repository (https://github.com/ikedamuneki/SteeringGA). All relevant data are available in our previous publication https://www.pnas.org/content/117/11/6178/tab-figures-data. We now mentioned them in Data Availability Statement (new page 21, line 490–493).

**Responses to the comments from Reviewer #2:**

*Ikeda et al., present an interesting study that nicely uses the advantages of mathematical modeling to disentangle whether the sinusoidal variation in sensory input imposed by the wiggling motion of the worm contributes to behavioral mechanisms for thermotaxis. They do this by developing 9 model worms using genetic algorithms and then testing how the worm responds to controlled sensory stimuli when aspects of motor response were turned off. They found that the models did not respond differently when sensory input was made sinusoidal. They also investigated how the activity of the model sensory neuron contributed to curving rates using singular spectrum analysis. This supported the idea that information transferred on the time scale longer than head bends is most important.*

*Major concerns:*

*The study tests a major hypothesis of whether the weathervane behavioral mechanism contributes to thermotaxis behavior in C. elegans, or just a version of the pirouette mechanism. Evidence for the weathervane mechanism has been found for*

*1. Ln 145. “on the scale of forward movement” and Ln 164 “scale of forward movement of worms” and other places – what exactly is this scale? It is not clear in this section what time scales and* *change-in-temperate rates you are comparing. Please explicitly explain these values side by side, and put in context with the durations of DV head bends and forward runs.* *Forward movement during runs has huge variation. Do you mean to conclude that the temporal scale important for causing steering* *maneuvers is X times longer than the duration of DV head bends?*

Yes, we conclude that the “temporal” scale of thermal input that causes steering maneuvers is longer than the temporal duration of DV head bends. We are comparing 1/4.2 Hz sinusoidal temperature change (**Fig 4B**) with monotonic temperature change (**Fig 4C**). These temperature changes assume thermal input sensed through head bends of worms that are moving perpendicularly to a thermal gradient of 0.5°C/cm and thermal input sensed through forward movement of worms that are moving parallel to the gradient, respectively. Considering that the velocity of head bends and forward locomotion are approximately 0.2 mm/sec, both change-in-temperature rates are estimated as 0.01°C/sec. As pointed out by the reviewer, forward movement during runs has huge variation, interrupted by turning and distorted by steering (new **Fig 1D**). Therefore, the monotonic temperature change in **Fig 4C** assumes the input sensed by worms that are moving straight up/down the thermal gradient without any turning nor steering for 20 sec. To make the above difference clearer, we conducted the following revisions:

Old page 7, line 145 – we add the word “temporal” (new page 7, line 148).

Old page 7, line 158 – we add the word “temporal” (new page 8, line 165).

Old page 7, line 160 – we add the phrase “of 1/4.2 Hz” (new page 8, line 166).

Old page 8, line 164 – we add the word “temporal” (new page 8, line 171).

Old page 8, line 165 – we add the word “straight” (new page 8, line 172).

Old page 8, line 165 – we add the phrase “without turning nor steering” (new page 8, line 172).

Old page 8, line 165 – we add the word “monotonic” (new page 8, line 173).

*2. If oscillatory input* *during DV head bends does not modulate curving rates, does it modulate pirouettes?*

We do not predict oscillatory thermal input during DV head bends can modulate pirouettes for migrating up/down a thermal gradient (i.e. positive/negative thermotaxis) since monotonic thermal input during forward movement would be also more appropriate for regulating frequencies of pirouettes. Instead, however, we predict that oscillatory input would be employed for moving isothermally at around worm’s cultivation temperature (i.e. isothermal tracking) (Hedgecock and Russell, 1975; Ikeda et al., 2020). A previous study showed that the amplitude of DV head bends during isothermal tracking were modulated not only by spatial but also temporal thermal gradients (Luo et al., 2006), implying that DV head bends can be responsible for isothermal tracking. We would like to attempt the implementation of isothermal tracking into the model circuit in our future study. We added above discussions in the paragraph 4 of the Discussion (new page 13, line 308–318).

*3. SSA correlation analysis provided one type of evidence that thermal info propagated from sensory neuron to motor neurons on a time scale longer than the DV head bending for the 9 models. It would be great to complement this correlational analysis with experimental analysis that modifies the networks on short and long time scales to independently test which time scale is most important and how. For instance, does filtering or adding noise on different time scales in the circuit interfere with performance? Does this support the idea that input on time scale of DV head bends doesn’t matter?*

Following the reviewer’s insightful suggestion, we added a different time scales of noise component to the model circuits (new **S11A Fig**) and assessed the effect on thermotactic simulations. The noise component is set to transmit oscillatory input to either AIY or DMN, and the frequency of the oscillation was changed from that of DV head bending to 10 times slower value. As shown in new **S11B Fig**, the applications of noise that oscillates with close frequency to that of head bending showed little or no impairments in the profiles of curving bias, whereas the applications of noise with slower oscillation impaired or diminished the curving bias toward higher temperature. We believe this result supports the idea that the transmission of activity from sensory to motor neurons on the longer time scale than head bending iscrucial for thermotactic steering behavior. We added above results in the section 5, paragraph 2 of Results (new page 11, line 256–262).

*Minor concerns:*

*A.) There are many places where the text would benefit from editing, and defining key terms and ideas as they are first introduced. These include:*

We thank the reviewer for the specific comments. We edited Abstract and the paragraph 3 of Introduction following the comments below.

*Abstract*

*Ln 21. Small animals, not small-size*

We replaced the phrase “small-size animals” with “small animals” (new page 2, line 23 and 39).

*Ln 21. Subtle *difference* in sensory input – what kind of “difference”? Temporal, spatial, or both? And/or modality? You don’t explain.*

We replaced the phrase “subtle difference” with “subtle spatial difference” (new page 2, line 23).

*Ln 28. Persistent thermal signals – it is critical to explain this better. Persistent in modality? Same modality but integrated across a period of time? How persistent? How much time? And persistent across time relative to what? DV head bends? And/or beyond? Context is important here.*

To clarify that we are focusing on persistence of temperature changes across “time” relative to DV head bends, we replaced the phrase “persistent thermal signals” with “temporally persistent thermal signals sensed beyond the time scale of sinusoidal locomotion of *C. elegans*” (new page 2, line 30–31). Also, we are assuming the persistence in “modality” of thermal signals and therefore used the phrases “persistent rise in temperature” and “persistent fall in temperature” in the subsequent sentence.

*Ln 29. Lessens steering rates – unless “steering” is defined otherwise later in the paper, readers will know that one can “steer” straight, so the wording is confusing. How about “turn rates”?*

We agree with the reviewer’s concern. Since we are using “turning” as another behavioral strategy in the manuscript (new **Fig 1D**), we instead replaced the phrase “steering rates” with “steering curvature” so that readers can imagine trajectories of worms (new page 2, line 30–34; new page 3, line48; new page 5, line97–103).

*Ln 29. *Persistant temperature increment* lessens.. this is awkward phrasing. Do you mean persistent changes in temperature? If so, then changes from what? Change and increment are all relative.*

Yes, we wanted to mean persistence in “temperature changes”. To avoid the impression of relativity in the wordings, we replaced the phrase “Persistent temperature increment” with “Persistent rise in temperature” and “temperature decrement” with “fall in temperature” (new page 2, line 31–33).

*Ln 171. …thermal input DURING the worm’s forward movement…*

We replaced the words “from” with “during” (new page 8, line 186–187).

*B.) Although no similarity was found for the values underlying the 9 models, it would be nice to hear how clustered the models were in search space.*

In new **S1A Fig**, we plotted the 32 independent parameter sets having a fitness score of at least 0.5, including 8 parameter sets having a fitness score of at least 0.6 which underwent subsequent analyses. Since the full dimension of search space is 23, we plotted the models in several subspaces: the space #1 defined by principal components of 6 bias terms that shift sensitivity range of inter- and motor neurons, the space #2 defined by principal components of 12 connection weights of chemical/electrical synapses, the plane #1 defined by a connection weight from to motor neurons and a connection weight of neuromuscular junction, and the space #3 defined by 3 terms that determine response property of a thermosensory neuron. We found the loose clusters of the models in the space #1 and in the plane #1, but they were not corresponding with each other. In our future study, we would like to investigate how good models evolve in search space and configure clusters.

**Responses to the comments from Reviewer #3:**

*In this study, Ikeda and colleagues used an evolutionary algorithm (EA) to identify neural network models that can drive sensory-gradient-guided steering behavior in simulated agents. The biological phenomenon that motivated this study was the thermotaxis behavior of C. elegans, which employs two key strategies: biased random walk (klinokinesis, akin to tumble-and-run in bacteria chemotaxis) and gradual steering during forward locomotion (klinotaxis). The authors used results from several experimental studies (in particular Ikeda et al. and Tsukada et al.) and the C. elegans connectome (White et al. 1986) to impose a priori constraints on the size, topology and functional properties of the model networks. Through in silico evolution, the authors identified 9 networks that yielded satisfactory fit to the thermotactic steering behavior in real animals. By examining these models, the authors concluded that the sole sensory neuron in the network, modeled after the thermosensory neuron AFD, functions by modulating the absolute magnitude of the curving rate, and does so over a timescale longer than that of the oscillatory locomotion of the (model) animal..*

*Overall, I found the approach of this study, namely analyzing computationally evolved networks in order to derive biological insights, quite interesting. However, I feel that the study could improve significantly on two fronts: 1) Provide a more mathematically precise and biologically motivated formulation of the network model and agent-based simulations. Importantly, if the goal of the study is to understand the circuit basis of steering/klinotaxis behavior, then the authors need to convince the reader that their evolutionary algorithm specifically targets the steering behavior, as opposed to generating alternative implementations of the biased random walk/klinokinesis. 2) The authors need to provide more substantial support for their main finding, namely that* *thermosensory inputs were transmitted from AFD to the downstream neurons at timescales slower than head oscillations (<=4 s per cycle). In particular, the authors need to convince the reader that this observation does not simply* *reflect* *the design of the thermal response kernel in AFD, which* *performs a temporal difference operation over a broad (~10 s) window.*

*SPECIFIC COMMENTS ON MODEL DESIGN/SELECTION:*

*1. I am not fully convinced that the evolutionary algorithm specifically targets the steering/klinotaxis behavior. According to the authors, models were selected based on* *goodness-of-fit to two key metrics: a) the temporal progression of the thermotaxis (TTX) index, b) the dependency of the absolute magnitude of the curving rate (𝜑) on movement direction. Firstly, since real animals perform both klinokinesis and klinotaxis during thermotaxis (Fig. 3C-E, Ikeda et al. 2020), the TTX index depends on a combination of these two behaviors. Since the simulated animals were also allowed to perform klinokinesis, it is unclear how much of the TTX profile result from klinokinesis alone. To specifically select for efficient klinotaxis behavior, the authors may need to reconsider the use of the TTX profile as a criteria.*

We understand the reviewer’s concern about whether our evolutionary algorithm specifically targets the steering behavior. To confirm the specificity, we have refined the formalization of goodness-of-fit (new **Fig 2C**), reperformed another 200 evolutionary searches (new **Fig 3** and **S1 Fig**), and replaced all the data in subsequent analyses. As pointed out by the reviewer, the regulations of both turning (klinokinesis) and steering (klinotaxis) (new **Fig 1D and 1E**) mediate thermotactic migration of *C. elegans*. In our previous study (Ikeda et al. 2020), the contribution of each of the strategy to the thermotactic migration (depicted by the temporal increase of TTX index) was quantitatively assessed by conducting thermotactic simulation (new **Fig 1F and 1G**). Therefore, in this study, we measured the difference between the TTX profile from model-based simulation (red line in new **Fig 2C**) and that from data-based simulation in which only the biased profile in truing frequencies was employed (magenta line in new **Fig 1G** and **Fig 2C**), yielding the new fitness value (new **Fig 2C**). We added above descriptions in the section 1, paragraph 1 of Results (new page 6, line 118–127) and in the section 3, paragraph 2 of Materials and Methods (new page 18, line 432–439). The reason why we did not formalize the fitness value as in new **Fig 2C** in our previous manuscript is that the non-evolved models showed absurd steering behavior and negated the contribution of biased turning to the thermotactic migration, resulting in no increase in TTX index. However, we think that the new formalization of goodness-of-fit is more convincing as an assessment of steering behavior. We thank the reviewer for the constructive comment.

*Secondly, a hall mark of klinotaxis behavior is the dependency of the exact curving rate on movement direction, which appears as a sinusoid-like function over a 2𝜋 period (see Iino and Yoshida, J Neurosci 2019). The authors have somehow chose to investigate the absolute value of the curving rate (|𝜑|). One risk looking at |𝜑| instead of 𝜑 is that its dependency on heading direction, which shows a peak at 𝜋/2, closely resembles the relationship between reversal (where |𝜑|≥150°) and heading directions (Fig. 3C, Ikeda et al. 2020). Thus, without putting a cap on |𝜑|, the evolutionary algorithm could potentially select models that excel at klinokinesis instead of klinotaxis. I could not find in the manuscript a pre-determined upper threshold for |𝜑|. I wonder what measure the authors have taken to ensure the selection is directed specifically at the klinotaxis behavior.*

As we now described in **Eqs 6–9** and in the section 3, paragraph 2 of Materials and Methods (new page 18, line 421), the upper threshold of $\left| \varphi\right|$ is determined by a parameter $w_{NMJ}$, which was evolved within the range from 0° to 90°. The resulting values of $w_{NMJ}$ in the 8 evolved models ranged from 20° to 86° (**S1 Table**), and the curving rates that were measured from the model worm’s trajectory were indeed within $w_{NMJ}$ (new **Fig 7A**). These ensure that the evolved behavior is distinct from turning behavior (new **Fig 1D**).

*2. In addition to the above, there is further confusion on what |𝜑| really is. Extrapolating from Fig 1D, |𝜑| is the absolute value of the change in the movement direction 𝜃, taken every 1s. Is |𝜑| calculated the same way in the model? While the periodicity of the head oscillation was not explicitly defined in the manuscript, it seems to be around 2-4 seconds based on Fig 8. This would explain why |𝜑| is oscillatory, as shown in Fig 7 and 8. However, in Fig 4-6 and many of the supp figures, |𝜑| does not oscillatory when plotted against AFD activity, temperature and/or heading direction. Is “|𝜑|" in these latter cases a smoothed version of the instantaneous* *|𝜑|? I see two associated terms “curving rate” and “curving bias” in the paper. Could the authors define them explicitly in the Methods?*

As the reviewer describes, $\left| \varphi\right|$ in old **Fig 7** and **Fig 8** was the instantaneous values calculated via the model (new page 16, line 371–374; new page 17, line 393–403), whereas $\left| \varphi\right|$ in old **Fig 4–6** was a smoothed version of the instantaneous $\left| \varphi\right|$. Since empirical $\varphi$ and $\theta$ were calculated from trajectories of the centroid of worms (Ikeda et al. 2020) (new **Fig 1E**), we smoothed the instantaneous $\varphi$ within 4.2 sec for eliminating oscillatory components. Although we had confirmed that profiles of curving bias $\varphi$ (new **Fig 1E** and **Fig 3C**) are not influenced by this smoothing procedure, we agree that different terms should be used for the individual values. Throughout new **Fig 4–8** in the revised manuscript, we analyzed only the smoothed version of $\left| \varphi\right|$ and named it as “steering curvature” $\Psi$ since it is not strictly the same formulation as the absolute value of $\varphi$. We now described the definition of “curving bias” $\varphi$ in the section 2, paragraph 2 of Materials and Methods (new page 17, line 393–403) and “steering curvature” $\Psi$ in the section 4 of Materials and Methods (new page 18–19, line 441–452). The term “curving rate” $\psi$ is now used for a temporarily defined value in the model as described in the section 1, paragraph 3 of Materials and Methods (new page 16, line 371–374).

*3. In describing their network model and agent simulations, the authors cite a number of previous studies without laying out the full set of equations and* *parametrizations (evolved or pre-defined). It would be greatly helpful to the reader if the author could present the full mathematical formulation of their model and agent simulations in the Method section. Examples of some questions I had while reading the methods:*

We now described the full set of equations for our circuit model (**Eq 1–10**) in the section 1 and 2 of Materials and Methods (new page 15–17, line 344–414) and parametrizations for evolutionary searches in the section 3 of Materials and Methods (new page 18, line 416–439).

*a. Which free parameters go into the nonlinearity ℎ for AFD? Assuming it is a sigmoid function, is the center of the sigmoid (shown at 17°C in the schematic) an evolvable parameter? Is so, is it surprising the all evolved models have a sharp drop in curving rate at around 17°C (Fig. S2)?*

The nonlinearity $h$ for AFD is a hill function, ant its formalization is now described in **Eq 2** (new page 15, line 352–356). We evolved three free parameters in $h$: $T_{thr}$, $K_{d}$, and $n$ within the range $\left[ 14, 26 \right]$, $\left[ 10, 100 \right]$, and $\left[ 1, 10 \right]$, respectively (new page 18, line 422–423). $T_{thr}$, which determines the foot of hill functions, evolved to approximately 15°C for migration up the thermal gradient (new **S1 Fig** and **S1 Table**) and approximately 21°C for migrating down the gradient (new **S4 Fig** and **S1 Table**). Therefore, the sensitivity range of AFD in the evolved models cover the temperatures around the center of the assay plates (17°C or 23°C), though how steering curvature $\Psi$ behaves within these sensitivity ranges is not obviously understood. Indeed, the newly evolved models in the revised manuscript exhibited a variety of profiles of $\Psi$ (new **S5 Fig**). We assume that whether the evolved models show a rise or a drop in steering curvature is determined by the combination of several factors including the definition of fitness value (new **Fig 2C**) and the shape of response property of AFD (new **S2A Fig**). We would like to measure the empirical profile of $\Psi$ and incorporate it in our future model.

*b. How are experimental data incorporated into the thermotaxis simulations? I assume that transitions rates between runs and various types of sharp reorientations were estimated from experimental data. Then what about in model ablation experiments shown in Fig. S1? Were the state transition rates taken from experiments on wild type animals or neuron-ablated animals?*

As the reviewer describes, experimental data were employed to determine transition rates between runs and turning; for every 0.1 second, simulated worms were decided whether to undergo an omega turn, a shallow turn, a reversal, a reversal turn, or a curve according to the experimental data of turning frequencies (Ikeda et al. 2020) (new **Fig 1E**). Besides, when the worms were decided to undergo turning, the next position $(x, y)$ and moving direction $\theta$ were determined according to the empirical probability distributions of the exit direction $\Phi$ after the individual turns. The experimental data of average displacements $(\Delta x, \Delta y)$ during the individual turns were also employed to decide the next position. For the simulations in **S1E Fig**, we employed all the data (turning frequencies, exit directions after turning, and average displacements during turning) taken from experiments on neuron-ablated animals. We now described above procedures in the section 2, paragraph 1 of Materials and Methods (new page 16–17, line 378–392).

*c. In the caption of Fig 2 (starting line 547), the authors state that “TTX index and curving bias from the simulations (red lines) and the experiment (black lines) were subtracted by those from the experiment without thermal gradients (gray lines).” Is it true that the experimental no-gradient TTX profile were subtracted from* *the simulated on-gradient TTX profile? Is it more appropriate to subtract a simulated no-gradient TTX profile instead?*

Yes, in the previous manuscript, we subtracted experimental no-gradient TTX profile from the simulated on-gradient TTX profile for calculating the fitness value (old **Fig 2C**). To answer the comment 1 above, we now calculated the fitness value in a different formulation; a simulated TTX profile based only on the biased profile in truing frequencies (new **Fig 1G**) was subtracted from the TTX profile in the on-gradient and model-based simulations (new **Fig 2C**).

*COMMENTS ON THE ANALYSES AND INTERPRETATION OF THE EVOLVED MODELS:*

*1. The main finding of the paper was derived from the analyses in Fig 8, which shows that AFD activity co-varies with a component of the curving rate that varies on the timescales of >4 seconds. As mentioned earlier, it is unclear if this phenomenon simply results from the difference in the time scales of the AFD thermal response kernel 𝑟 and the frequency of the head oscillation. Could the authors perform in silico experiments to test this possibility?*

To investigate whether our main finding is solely depending on the design of the thermal response kernel $r$ in AFD, we performed additional evolutionary searches in which the response kernel was replaced with that of another sensory neuron AWC to a chemical stimulus (Kato et al., 2014) (new **S2A Fig**). This alternative response kernel performs a temporal difference operation over a narrower window that of AFD. As shown in new **S7B Fig** and **S10A and S10C Fig**, the main finding of the paper were verified in the evolved models; the steering curvature of the model worms were modulated based on monotonic thermal inputs, and thermosensory inputs were transmitted from AFD to the downstream neurons at timescales slower than head oscillations.

We further attempted evolutionary searched in which the response kernel was replaced with two-times compressed $r$ in temporal axis. Across 100 searches, however, we obtained no parameter sets having a fitness score of at least 0.1, suggesting that the temporal operation of the response kernel is crucial for reproducing the empirical thermotactic behavior. Alternatively, we performed additional evolutionary searches in which wave period of a pattern generator CPG ($t_{OSC}$) was evolved within the range $\left[ 0.5, 50 \right]$ sec, thereby giving models an opportunity to employ thermosensory inputs through slower head oscillation for modulating steering curvature. Even in the models with two-times (or more) slower head oscillations, however, thermosensory inputs were transmitted at much slower timescale (**S10B and S10C Fig**). These results show that the design of the thermal response kernel in the previous manuscript was not solely responsible for the main finding the paper. We added above results in the section 5, paragraph 2 of Results (new page 11, line 254–257)

*2. As mentioned above, the defining feature of klinotaxis is the animal’s ability to gradually modify their heading direction to point up (in the context of positive chemotaxis) the gradient direction. By definition, the sign of the curving angle is crucially important, because it could either orient the animal towards or away from the desired movement direction. In contrast, the selected models were evolved to perform a gross modulation of the magnitude of the curving rate |𝜑| based on heading direction and they indeed do so. As the authors pointed out in Discussion, it seems that the solution these models arrived at is one of klinokinesis instead of klinotaxis, whereby the agents* *vigorously reorient themselves until settling on a trajectory that efficiently brings them up the thermal gradient. I think this relates to my earlier comment that the evolutionary algorithm may not be specifically targeting the steering behavior (for which the sign of 𝜑 is crucially important), but may have developed a slower version of the biased random walk/klinokinesis. This distinction is important if the authors wish to propose that they have identified a potentially novel strategy for thermotaxis.*

As we now described in the section 1–3 of Materials and Methods, the model worms regulate the signed curving rate $\psi$, not the absolute value of it (**Eq 6**). Since the sign of $\psi$ is determined by the difference in activities of dorsal and ventral neck motor neurons, $\psi=+\varphi$ when the dorsal side of the model worm is toward the warm direction, and $\psi=-\varphi$ when the ventral side is toward the warm direction (**Eqs 8 and 9**). Therefore, models have an opportunity to be evolved to directly modulate $\varphi$ and gradually modify their heading direction to point up warmer direction based on thermal input sensed through head swings (**S6A Fig**). However, in our evolutionary searches, models were evolved to modulate the magnitude of the curving rate and vigorously reorient themselves until pointing up wormer direction based on thermal input sensed through forward movement (**S6B Fig**). The important point is that within previous studies on *C. elegans* behavioral analysis, biased profiles of $\varphi$ as in new **Fig 1E** have been regarded as an evidence of the implementation of klinotaxis. By contrast, in this study, such biased profiles were shown to be reproduced by the klinokinetic regulation of steering behavior (**Fig 3C** and **Fig 5C**). These results do not deny the existence of klinotactic regulation during thermotaxis but demonstrate the potential implementation of klinokinetic steering, which has not been recognized previously. Further, we are assuming the possibility that the chemotactic steering behavior is also executed klinokinetically. These possibilities would be verified in assays in which thermal/chemical stimuli are temporally applied, and worm behavior is monitored simultaneously, as performed in **Fig 4**. We added above discussions in the paragraph 3 of Discussion (new page 13, line 297–308).

*3. In line 197, the authors claim as a new finding that “**Activity of a thermosensory neuron decides curving rates of locomotion”. Since AFD is the sole sensory neuron in the model network, and given that the models were selected based on their ability to modulate curving rates, wouldn’t it be obvious that any temperature-dependent modulation of the curving rate has to come through AFD?*

As the reviewer points out, the previous subheading did not represent a new finding in the section. We now replaced the sentence “Activity of a thermosensory neuron decides curving rates of locomotion” with “Higher activity of a thermosensory neuron induces straight movement of worms” (new page 9, line 211).

*4. Lastly, the dynamics of neural activity in Fig 7 and of the curving rate decomposition in Fig 8 were plotted as heat maps, which were hard to decipher. I would suggest replotting them as simple line plots.*

We replotted the heat maps in **Fig 7A** and **Fig 8B** as the line plots.
